# Twenty-three-year demographic history of the Affenberg Japanese macaques (*Macaca fuscata*), a translocated semi-free-ranging group in southern Austria

**DOI:** 10.1007/s10329-021-00928-4

**Published:** 2021-07-10

**Authors:** Lena S. Pflüger, Katharina E. Pink, Bernard Wallner, Claudia Radler, Markus Dorner, Michael A. Huffman

**Affiliations:** 1 Department of Behavioral and Cognitive Biology, University of Vienna, Djerassiplatz 1, 1030 Vienna, Austria; 2 Austrian Research Center for Primatology, Ossiach 16, 9570 Ossiach, Austria; 3 Family and Population Studies, KU Leuven, Parkstraat 45, 3000 Leuven, Belgium; 4 Institute of Zoology, University of Graz, Universitätsplatz 2, 8010 Graz, Austria; 5 Affenberg Zoobetriebsgesellschaft mbH, Ossiach 16, 9570 Ossiach, Austria; 6 Primate Research Institute, Kyoto University, 41-2 Kanrin, Inuyama, Aichi 484-8506 Japan

**Keywords:** *Macaca fuscata*, Demographic history, Translocation, Reproduction, Mortality, Life expectancy, Social dynamics, Rank relations

## Abstract

Demographic studies on translocated primate groups provide a unique opportunity to study population dynamics, social strategies, and reproductive parameters of a species adapting to new environments. In 1996, 38 Japanese macaques (*Macaca fuscata*) of the Minoo-H group (Osaka Prefecture, Japan) were translocated to Affenberg Landskron, a four-hectare naturally forested park in southern Austria. By January 2020, the population had increased to 160 individuals, and a total of 223 births were recorded. Births peaked in late April to late May, and the timing was influenced by neither offspring sex nor parity status of the mother. Infant mortality was low (8.97%), mostly involving primiparous females, and the average interbirth intervals were shorter following the death of an infant (1.10 years) than a surviving infant (1.77 years). Females rarely had offspring with the same males repeatedly, and the reproductive success among males declined with increasing years of presence in the group. The main aspects of reproduction, mortality, and mate choice are consistent with published data on natural and provisioned populations in Japan and those translocated to other countries. The life expectancy for females, however, was relatively high (11.72% chance of reaching the age of 20), whereas birth control prevented them from using their lifetime reproductive potential. By January 2020, the number of old individuals (> 18 years; 17.5%) was close to that of juveniles (< 4 years; 22.5%). The specific group composition, along with the inability of males to emigrate out of their natal group, may affect the social dynamics of the population, which merits further attention in future studies.

**Supplementary Information:**

The online version contains supplementary material available at 10.1007/s10329-021-00928-4.

## Introduction

Assessing the long-term demographic characteristics of translocated primate groups provides a unique opportunity to better understand many aspects of a species’ behavior and ecology as well as its ability to adapt to a new environment (Chalmers et al. 2012; Fedigan et al. 1983; Koyama et al. 1992). Japanese macaques (*Macaca fuscata*) are considered to be highly adaptable because they are the most northern-living non-human primate species, found across subtropical and temperate regions including areas of extreme cold and heavy snowfall. They have been the subject of long-term investigations not only across their natural distribution (e.g., Baldwin et al. 1980; Huffman 1991a; Huffman et al. 2012; Ikeda 1982; Maruhashi 1982) but also in quite different habitats outside of Japan to which they have been translocated (Fedigan et al. 1983; Gouzoules et al. 1981; Huffman et al. 2012; Pflüger et al. 2014).

In August 1996, a group of 38 Japanese macaques was translocated to Landskron, Carinthia, in southern Austria. The macaques were captured from the free-ranging Minoo-H group (Kawamura 1958; Leca et al. 2014; Yamada 1963), located near Minoo City, Osaka Prefecture, Japan (latitude 34° 40′ 10.3044″ N; longitude 135° 29′ 49.2324″ E). Information on the original group in Minoo-city was provided by a Japanese expert team who supervised the translocation from Japan to Austria. After 12 days of quarantine to undergo medical checks, the group was released into a four-hectare enclosure of natural forest surrounded by an electric fence. In 1997 the site was officially opened as a touristic monkey park called *Affenberg Landskron* (Monkey Mountain Landskron).

For the last 23 years, basic demographic data (birth, death, injuries) and information on provisioning, birth control, male social rank, and paternity have been collected as part of the management and scientific research protocols. The present study analyzed these data to draw a detailed demographic picture of the only population of Japanese macaques to be translocated to Austria. Our study provides insights into their habitat, population dynamics, reproduction, mortality, and social parameters. The comparison of our data with published data from other groups of Japanese macaques adds to our understanding of this species and provides essential background information for future studies on this and other groups of Japanese macaques in- and outside of Japan.

## Methods

### Study site and subjects

#### The habitat

The Affenberg Landskron is located at the top of a hill near the Landskron castle ruins in a rural area approximately 10 km from the city center of Villach, Carinthia (latitude 46°38′32.51″ N; longitude 13°53′48.98″ E).

**Fig. 1:**
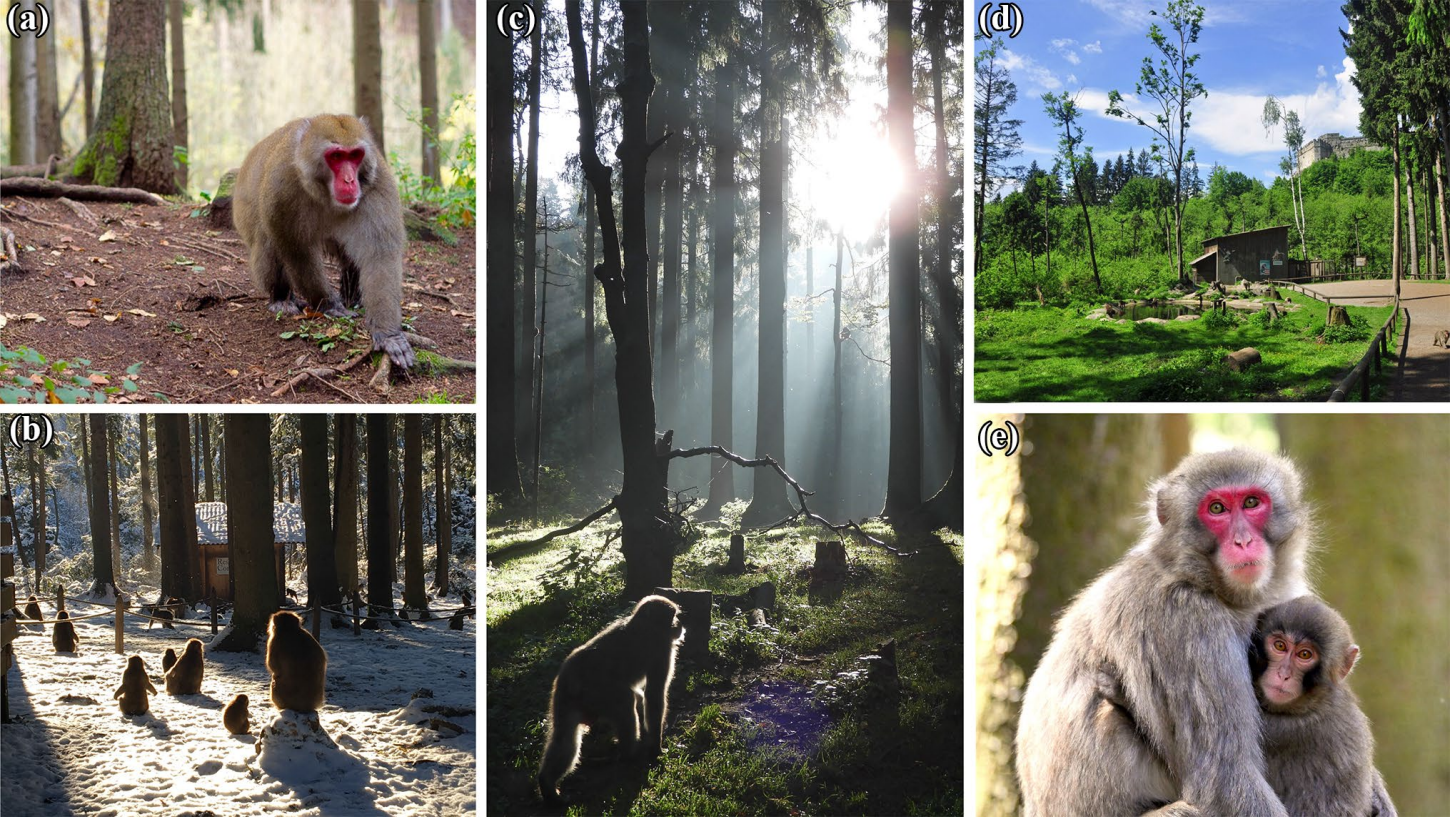
The Japanese macaques (*Macaca fuscata*) of the Affenberg Landskron, Carinthia, Austria, in their 40,000 m^2^ forest enclosure. **a** Adult male in autumn, **b** sunbathing during winter, **c** adult female in the morning sun, **d** center part of the enclosure and monkey hut (medical care station) during summertime, **e** adult female with 1-year-old juvenile. Photos Konstanze Meindl, Lena Pflüger, Pia Böhm, Affenberg Landskron, Klaus Freithofer, respectively.

The vegetation of the 40,000 m^2^ monkey enclosure is a natural mixed forest common to southern Austria, including *Picea abies*, *Pinus sylvestris*, *Salix* spp., *Poaceae*, *Sambucus nigra*, *Corylus avellana*, *Rubus idaeus*, *Urtica* spp.*, Petasites* spp., and *Oxalis* spp. The dominant species is tall spruce (*P. abies*), reaching a height of up to 30 m. A small natural stream runs through the enclosure. For descriptive purposes, the area is roughly divided into a central and a peripheral section (see electronic supplementary material Fig. S1). The central area consists of a rather sparse forest with clearings of grassland and bare ground under the spruce trees (Fig. 1). A 2-m-wide and 350-m-long visitor pathway leads through the central area (see electronic supplementary material Fig. S1). The periphery consists of a small, forested slope with spruce trees as well as densely vegetated lower strata of shrubs and swamp areas. At the outer edge of the peripheral section is a 6-m-broad grassland and gravel buffer zone leading up to a 3.3-m-high electric fence topped by a Perspex plastic sheet (1 m in height) for additional security to prevent monkeys from climbing out.

Carinthia has a Central European climate with four distinct seasons, including moderately wet but hot summers. In winter, the area is frequently covered with snow and the temperature often drops below freezing. Due to long and harsh winters, Carinthia is considered a moderate temperate region in Austria, with a yearly mean temperature of 9.56 °C (SD 0.67 °C). The mean temperature in spring (March–May) is 10.14 °C (SD 0.87 °C); summer (June–August) 18.21 °C (SD 0.73 °C); autumn (September–November) 9.51 °C (SD 0.97 °C); and winter (December–February) −0.90 °C (SD 1.42 °C). For more information on climate conditions also see Pflüger et al. (2019).

#### The park management

The Affenberg Landskron is a tourist facility where visitors are allowed to enter the enclosure on a guided tour only. The park is open annually for tours from 1 April to 2 November. During winter, the contact with humans is limited to one daily scheduled feeding session by the animal care staff. Researchers have full access to the population year-round, subject to permission from the park management, and the research they can conduct is limited to noninvasive studies.

In the early days of the park’s operation, the management took advice from primatologists regarding food provisioning and birth control to ensure optimal management conditions of the newly arrived group. Over the years, the facility has established scientific collaborations with different national and international research partners. In 2019, it became a field research station of the University of Vienna.

#### Individual identification and monitoring

The monkeys in Affenberg have never been tattooed or marked for individual identification. Therefore, the Affenberg team learned to identify each monkey via individual facial and body features and gave each of them a name. New staff and students were trained by core members on individual recognition and care protocols. Except for feeding, veterinary care, or sterilization, no person was ever allowed to touch or interact with monkeys or to otherwise interfere in their daily lives. Each day, trained staff monitored the group in the morning during feeding times, walking throughout the central and peripheral areas (see electronic supplementary material Fig. S1). During these inspections as well as during guided tours, the staff would routinely record any births and deaths detected. The staff was trained to identify anomalies in body functions and behavior (injuries, lethargy, body shivering, behavioral changes, motoric constraints, loss of appetite, etc.) to be able to report those to the veterinarian.

#### Veterinary care

A veterinary care station, installed in the center of the monkey enclosure, has been available for veterinary treatment since the monkeys arrived in 1996. After arrival, each monkey went through a health check-up, including treatment with doramectin for gastrointestinal parasites, a tuberculosis test, and rabies vaccination in the station before being released into the forest enclosure. Thereafter, veterinary care was limited to (1) the injection of an identification chip (transponder) at several months of age, (2) tubal ligations for sterilization, and (3) the treatment of severe injuries and infections. All treatments were performed by a veterinarian specialized in wild animal veterinary medicine. Neither vaccinations nor regular preventive examinations were administered. The health condition of the animals was monitored daily by caretaker staff during feeding times when individuals gathered at designated feeding spots.

#### Provisioning

The monkeys forage on the natural vegetation, which provides a variety of leaves, seeds, bark, roots, flower buds, and insects (for details see “The habitat”). Nonetheless, the amount of natural vegetation is limited, especially during winter months, and to ensure that the vegetation inside the enclosure remains intact, the monkeys are provisioned throughout the year. The daily amount of food provided was determined based on data records on the daily energy expenditure of free-ranging groups of Japanese macaques living in cool temperate areas with snow cover (Hanya 2003; Hill 1997; Jaman et al. 2010; Nakayama et al. 1999). The values were regularly adjusted according to fluctuations in group size. About 900 kcal/day for adolescents and adults and 400 kcal/day for juveniles (under 3 years) were provided on a daily basis. This equaled 8–10 kg of wheat, 80 kg of apples, 20 kg of carrots, and 60 kg of potatoes daily at the time of the present study (160 individuals, January 2020). In spring, the amount was reduced (about 20% less kcal/day) and was again resumed in autumn. To avoid monopolization by high-ranking individuals, food is widely distributed throughout the enclosure, including the periphery.

#### Birth control

Since the group’s arrival at Affenberg, the entire social group has ranged freely within the enclosure. To prevent overcrowding, selective birth control has been performed since 2000. To avoid interfering with the natural menstrual cycles of females, hormonal contraceptives were not used. Depending on female kinship and the size of matrilines, females were sterilized by tubal ligation after they gave birth to at least one offspring. Particular attention was paid to maintaining sexually intact females per matriline to protect each female lineage from extinction.

### Available data on the founder group

A document delivered together with the group in 1996 outlined the genealogy of the original Minoo-H group (dated 31 March 1995). It shows the names (in Japanese), birth dates, and kin relationships (mother–offspring) of the original group members native to Minoo, Osaka (*N* = 89). According to this phylogenetic tree, the Minoo-H group was originally formed from three matrilines and included a total of 11 adult males, 25 adult females, 22 immature males, 27 immature females, and four individuals of unverified sex. Of these animals, 38 were translocated to Austria in 1996 as the founders of the Affenberg group (13 sexually mature females [4–16 years], 10 immature females [1–3 years], 15 immature males [1–4 years]); no adult males were brought to Austria (for age and sexual maturation in Japanese macaques see Wolfe 1978). For each of these individuals, only the respective year of birth was available, so they were registered as being born on 1 January.

No information on the relatedness of the 38 translocated animals was provided when they arrived in 1996. They were not identified in the Minoo-H group kinship records, and no genetic analyses were performed at that time. Approximations of kin relationships could therefore be made only for six mature females who were accompanied by a juvenile younger than 2.5 years upon arrival. Those juveniles (*N* = 3 females; *N* = 3 males) were assigned by the Affenberg as offspring of those females upon their arrival in Austria. Two immature individuals (a 3-year-old female and a 2-year-old male) were assigned as siblings based on their patterns of proximity to each other. All other females who showed high levels of avoidance were considered to be unrelated and were assigned to separate matrilines. Except for three males (not older than 2.5 years), who were in close proximity with a mature female from the onset, males could not be tentatively assigned to any matriline. Hence, the remaining males (*N* = 12, between 1 and 4 years) were treated as unrelated to any of the arriving females, although the possibility that they were natal to the founder group cannot be entirely ruled out. Females categorized as unrelated represent the starting point of each of the matrilines still present today. A student thesis on network analyses on those females and their relatives conducted in 2011/2012 supports the initial assignment made by Affenberg in 1996 (Werdenig 2014). The founder females and their genealogies are listed in the electronic supplementary material Fig. S2.

### Longitudinal records

The recognition of individuals via daily inspection rounds (see above) has enabled written records to be maintained by the animal care staff to document births, deaths, injuries, medical treatment, and kin relationships of each individual since the group’s arrival in 1996. The present study has access to these records along with long-term personal observations and behavior records provided by the Affenberg staff members and associated researchers/students. The data set covers a period of 23 years, starting from the group’s arrival in August 1996 until 1 January 2020.

#### Birth records

Although parturition itself has not been witnessed, females with their newborns returned to the center of the group shortly thereafter. Newborns could be assigned to their mothers because remains of the natal cord were usually still attached, and bloodstains were visible on the mother’s fur. Furthermore, the Affenberg staff maintained records on female age and sterilization status, so it was known which female was expected to give birth. Births were registered as soon as a female was observed carrying a newborn, irrespective of whether the offspring was alive, dead, or died shortly after birth. Abandoned offspring (either dead or alive) found in the enclosure were also recorded. Miscarriages could not be considered.

#### Death records

The Affenberg records note the date of death in the following cases: (1) actual date of death when the death of an individual was observed, e.g. the animal was found still alive in the enclosure but died shortly thereafter under observation, or the animal died during veterinary treatment or nursing in the animal care station; (2) date discovered dead when an individual’s body was found, not decomposed, and the individual could still be identified; or (3) date last seen alive when an individual disappeared and was not found dead or alive after intensive search. In six cases (*N* = 5 males, *N* = 1 female), the Affenberg records note an individual to be last seen in the end of a certain year. Here, we used 31 December as the date of death for analytical purposes.

### Data processing and analyses

#### Group size and composition

Data records on births and deaths are used to assess the annual group size and composition during the entire study period. The annual growth rate was assessed each year on 1 September as the percentage difference in total group size between two subsequent birth seasons taking birth and death records of each foregoing year into account (see electronic supplementary material Table S1).

#### Socionomic sex ratio

The socionomic sex ratio (SSR) was calculated as the ratio of sexually mature males (≥ 4.5 years) to sexually mature females (≥ 3.5 years): $\frac{{\rm mature males}}{{\rm mature females}}$ × 100. The annual SSR was assessed at their arrival in August 1996 and in the beginning of each subsequent year (1 January) thereafter to 1 January 2020.

#### Paternity

Paternity analyses were conducted between April 2009 and February 2012 by the Karl-Franzens University of Graz, Austria, using hair samples as the DNA source (Radler 2014).

#### Social status

Information on male social rank was provided from previous research results using established protocols for behavioral observations of dyadic interactions between males collected in the mating period of 2012 (Pflüger et al. 2014, 2016), 2015, and 2016 (Herzele 2017). In these studies, dominant (e.g., chase, threat, spatial displacement, physical attacks) and submissive (e.g., flee, fear grin, avoidance) behaviors were used to assess the proportion of encounters “won” by an animal against another in a pairwise interaction (Singh et al. 2003).

In the earlier years, no detailed behavior records on dominance interactions were made. Student reports, however, name the identity of alpha, beta, and gamma males (Radler 2014; Werdenig 2014). These reports agreed with personal observations by long-term staff members of the Affenberg and, for those males who were still alive, with data from Pflüger et al. (2014, 2016). No information on female social rank was available from previous studies. Therefore, only a rough classification of matrilineal rank could be made with the consensus of experienced students who conducted behavioral observation during winter 2019/2020 and long-term staff member input. The estimated rank order of female matrilines ranging from 1 (highest-ranked matriline = “Esther Line”) to 17 (lowest-ranked matriline “Tanja Line”) is given in the group genealogical chart (electronic supplementary material Fig. S2), but the information was not used for any analyses in the present study.

#### Birth records

Birth records were analyzed from 1997 (first birth at the Affenberg) until 2019, the last covered birth season of the present study. Abandoned offspring (either dead or alive) found in the enclosure were also included in the analyses in terms of the total number of births at Affenberg. Motherhood was assessed for all females who gave birth. One abandoned and hand-raised newborn (“Fanny”) was excluded because the mother’s identity could not be determined. Age at first birth, as well as primiparous status, was assessed only for those females who reached sexual maturity at the Affenberg (< 3.5 years old at time of arrival); mothers who arrived sexually mature in 1996 were excluded in these analyses (*N* = 11). Multiparous status was assessed for all females who gave birth multiple times at Affenberg. Three births had to be excluded due to unknown birth order. In these cases, the mothers were 4 or 5 years old at the time of arrival in 1996, and their parity status could not be assessed.

Reproductive potential of females was assessed by the percentage of females who actually gave birth at the Affenberg out of those who were reproductively intact during the preceding mating period (sexually matured ≥ 3.5 years and not yet sterilized).

Interbirth interval (IBI) was calculated as the time interval (in days) between two subsequent births. Overall IBI, IBI after infant survival/death, and IBI after live-born male/female were assessed for all females with multiple births at Affenberg. Those females who arrived already sexually mature (≥ 3.5 years) in 1996 were excluded (*N* = 11) from the analyses regarding the IBI between first and second birth as well as the IBI between second and third birth because parity status could not be assessed.

The yearly sex ratio at birth (SRB) is the ratio of males to females born: $\frac{{\rm male\, births}}{{\rm female\, births}}\times100.$ This provides insights into the sex composition of this population. The sex could not be determined for three offspring who died and disappeared shortly after birth. Although those individuals could be assigned to a mother, they had to be excluded from the analyses in terms of SRB.

#### Mortality and age range

Life span was assessed using the Affenberg longitudinal records on birth and death dates as described above. Those records also include the founder individuals born in Japan (*N* = 38), whose date of birth was registered as 1 January of the respective birth year (see section “Available data on the founder group”).

### Data preparation and statistical analysis

All statistical analyses were performed in Stata 14 software and Microsoft Excel 16.50.

## Results

### Social and demographic parameters

#### Matrilines

The Affenberg population currently includes 11 intact matrilines ranging from 5 to 16 individuals including juveniles. Five of the original female lineages are represented by only two or fewer females. Four original females died before they could give birth (electronic supplementary material Fig. S2). One additional matriline emerged in 2010 when a newborn female, “Fanny,” was found by the animal care staff abandoned in the enclosure. Since none of the females showed interest in her, she was hand-raised and returned to the group at 8 months of age. After being rejected by female group members, she was alternately accompanied (travel together, co-feeding, grooming), carried (on back and belly), and supported by two adult males, two brothers, named “Stefan” (9 years old) and “Karl-Vinzenz” (10 years old). Stefan died in 2012, but Fanny has maintained social contact with Karl-Vinzenz until today. After Fanny reached sexual maturity, she was repeatedly seen in consorts with other males, but no sexual behavior has ever been observed between Fanny and Karl-Vinzenz. Paternity analyses (Radler 2014) excluded both Karl-Vinzenz and Stefan as the potential father of Fanny. Two possible mothers were detected, making Fanny assignable to either the Nicki or the Anastasia kin group. In 2015, Fanny gave birth to a daughter. Together with her daughter she represents her own separate matriline in the Affenberg group. For more information on the origin and current size of each matriline see electronic supplementary material Fig. S2.

#### Group size and composition

After a loss (−5.26%) in the first year following arrival, the group had already recovered 1 year later, showing the highest growth rate (25%) ever recorded in the group (electronic supplementary material Table S1). At the time of the present study (1 January 2020), the group consisted of 160 individuals.

**Fig. 2:**
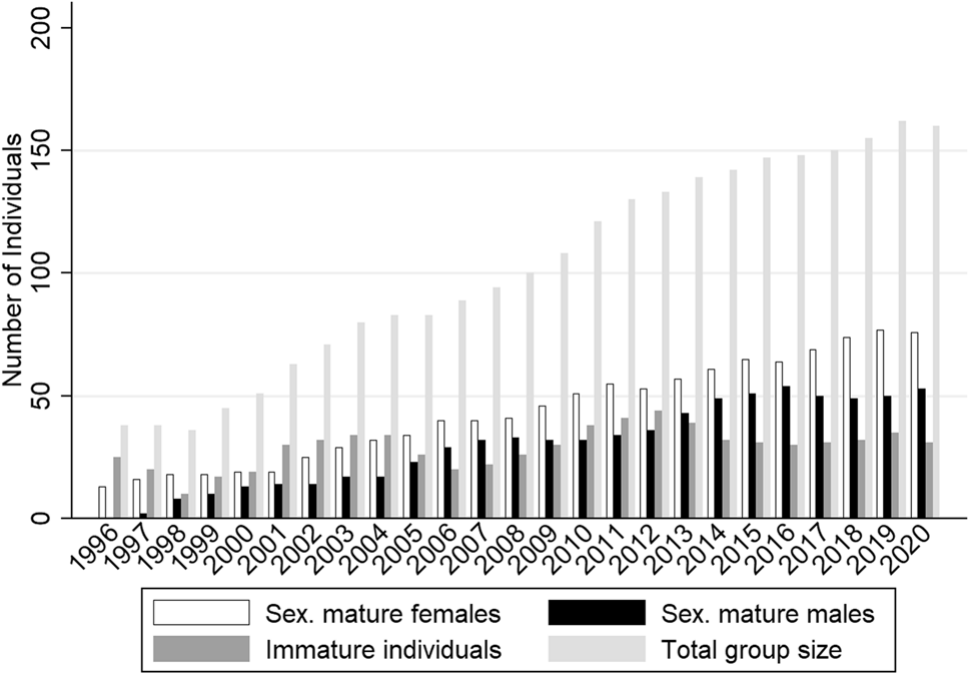
Yearly change in group size and age-sex class composition

Over the years, the group composition has comprised on average 41.61% sexually mature females (≥ 3.5 years), 26.44% sexually mature males (≥ 4.5 years), and 31.95% immature individuals. The highest proportion of mature females was present in 1998 (50%), the lowest in 2001 (30.16%). No sexually mature males arrived in Affenberg in 1996. The following year, the percentage of mature males increased to 5.26%, and reached 22.22% in 1998. The proportion of mature males was highest in 2016 (36.49%). The percentage of immature individuals was highest in the year of arrival (65.79%) and lowest in 2020 (19.38%). In January 2020, the group composition (*N* = 160) included 47.5% mature females, 33.13% mature males, and 19.38% immature individuals (Fig. 2).

**Fig. 3:**
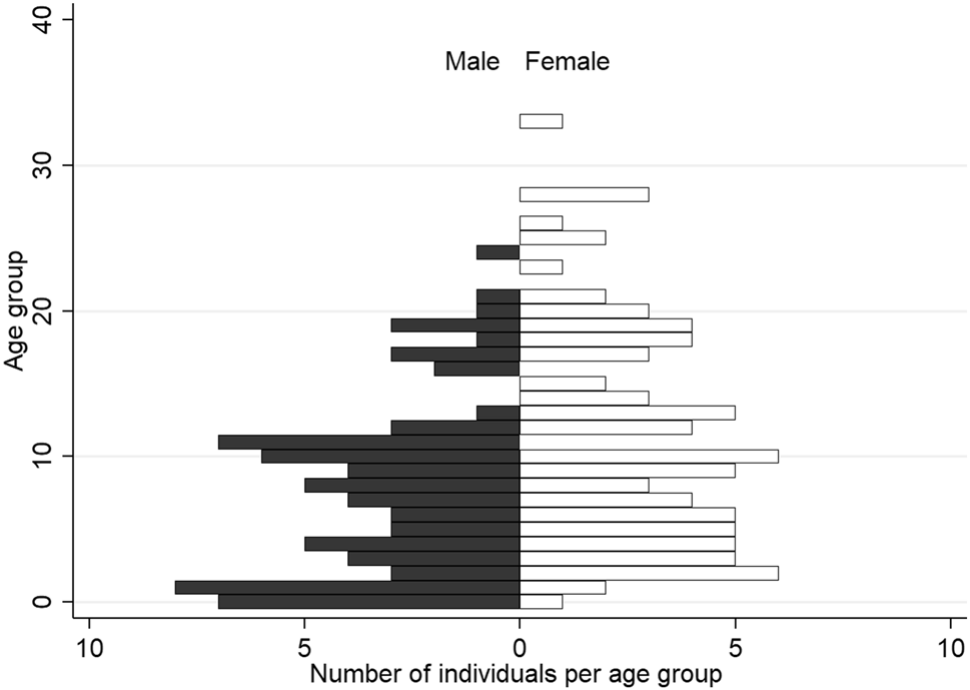
Current population pyramid cutoff date 1 January 2020

Upon arrival in 1996, the group consisted of 60.5% individuals classified as “young” (< 4 years) and none as “old” (> 18 years). The oldest arrival was a 16-year-old female. On 1 January 2020, the group included 22.5% young and 17.5% old individuals. The age-related structure of the group depicted in Fig. 3 reveals that females have a surplus in the age groups 10+ years. Males are overrepresented in the age groups 0–1 years and 1–2 years.

#### Socionomic sex ratio (SSR)

Aside from the first year, in which no mature males were present, the annual SSR ranged from 12.5 to 84.38 (mean: 63.08; SD 19.69). The exceptionally low SSR value of 12.5 occurred in 1997 when the group comprised 16 sexually mature females and only two sexually mature males. The SSR peaked in 2016 (84.38), when the number of mature males (*N* = 54) was comparable to the number of mature females (*N* = 64).

#### Stability of alpha status

The first reliable information on female rank status dates back to 1999 when “Esther” was recognized as the alpha female. Esther was the oldest female at the time of arrival (16 years old) and was the first to give birth at Affenberg in 1997. Her second and only female offspring, Luise, was born in 1999. Three years later Esther died. Her daughter Luise gave birth to her only offspring, a male, who died at the age of four. Although her matriline disappeared, Luise has maintained her inherited alpha female status within the group up to the time of this analysis.

**Fig.4:**
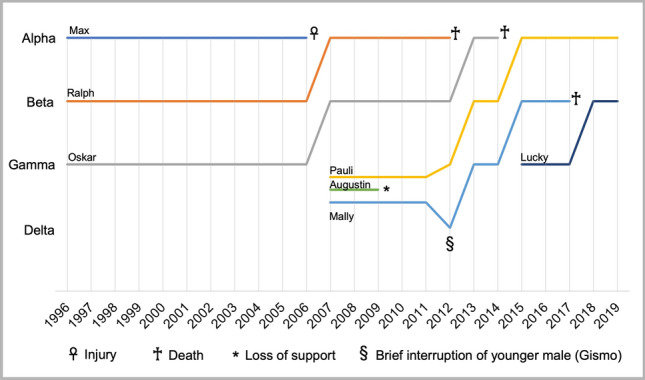
Change in social rank position, tenure lengths, and cause of status loss of top-ranked males.

Within the 23 years of this study (cutoff point 1 January 2020), alpha and beta male status has changed four times, each time without aggressive takeovers or reshuffling of the leading positions (Fig. 4). The first alpha male was recognized in autumn 1996: “Max,” 4.5 years at that time, was the first alpha male, followed by “Ralph” and “Oskar” holding beta and gamma positions, respectively. Max maintained alpha status until he was isolated for 6 weeks due to medical treatment for a bone fracture in November 2006. After his recovery and subsequent release, he withdrew into the periphery and kept his distance from the group until his death in November 2014. The beta male Ralph (14 years) assumed the alpha position when Max was removed for medical treatment. With Ralph’s rise to the alpha, Oskar (13 years) became the beta male. The opened gamma position was not as easily filled. Two males expected to be next in rank, Pauli (6 years) and Mally (8 years), struggled with another male, “Augustin” (5 years), who aggressively contested the position (Radler 2014). After about 2 years, Augustin lost social support by challenging Pauli and Mally for rank (his attempt was not supported by other group members) and settled at a lower rank position (Radler 2014). Dominance analyses conducted in 2012 assigned Pauli (12 years) as gamma and Mally (14 years) as delta male, with Augustin in the 16th-ranked position (out of 30 males; Pflüger et al. 2014). Based on the rank scores, however, Mally shared his position with the 11-year-old male “Gismo,” who reached comparable rank scores. Only several months later, the alpha Ralph (20 years) died, and Oskar (19 years), Pauli (12 years), and Mally (14 years) took over the alpha, beta, and gamma positions, respectively. Gismo had lost his status and died 1 year later. After Oskar died in 2014, Pauli (14 years) became the alpha and Mally (16 years) the beta male. The gamma position was filled by a young male, “Lucky” (8 years old). Mally died in 2017 (19 years), leaving Lucky (11 years) in the beta position. Up to the present, Pauli (19 years) and Lucky (13 years) have maintained their respective rank positions (Fig. 4).

### Birth records

#### Female reproductive potential

Female reproductive careers were influenced by tubal ligation (sterilization). A total of 76 females were sterilized during the 23 years of this investigation (mean age at sterilization: 8.58 years; SD 3.23 years). Due to birth control (or early death, *N* = 18), no female has reached her full reproductive potential (the oldest female to be sterilized was 20 years, followed by a 16-year-old female). The reproductive window of those who reached sexual maturity (≥ 3.5 years, *N* = 86) ranged from less than 1 year to almost 17.5 years (mean: 4.89 years; SD 3.25 years). For an overview on the annual number of sterilized females and fertile females, see electronic supplementary material Table S2.

Not all sexually mature, reproductively intact females reproduced every year (Wallner et al. 2011). On average only 51.03% (SD 12.46%) of these potentially fertile females (PF) gave birth to an infant in the subsequent year. In the present study, reproductive potential peaked in 2002, when 63.64% of the PF females gave birth. In the first birth season after arrival at Affenberg, in 1997, only 6.25% of PF females successfully gave birth. One year later, 55.56% of those females gave birth. Thereafter, interannual variation (see electronic supplementary material Table S2) was unrelated to either the increasing group size or the availability of sexually mature males (data not shown).

#### Age of mothers and birth timing

**Fig. 5:**
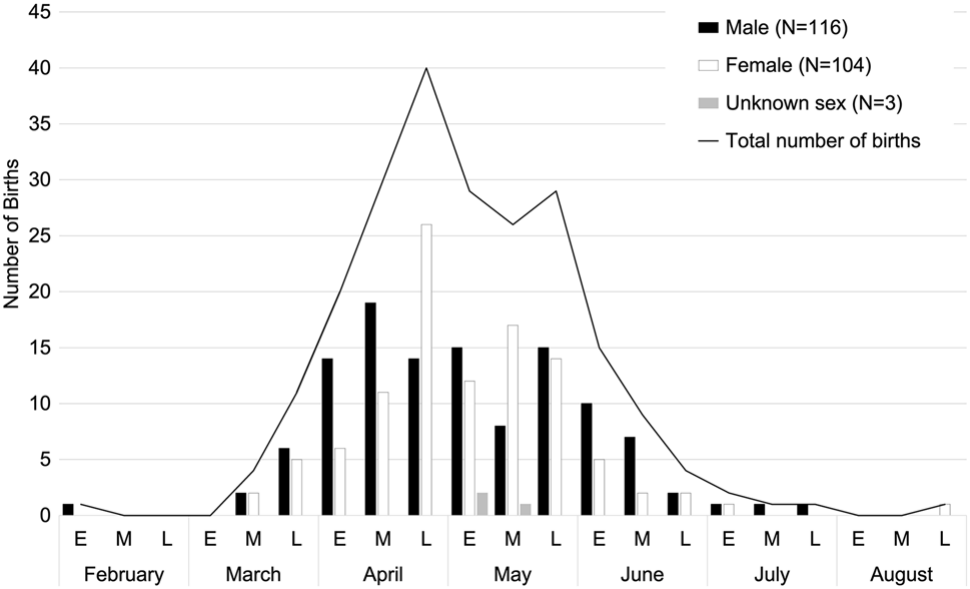
Monthly distribution of all 223 births recorded between 1997 and 2019 according to sex. Each month is divided into trisections: E: early; M: middle; L: late

Over 23 years, a total of 223 births were recorded at Affenberg (104 females, 116 males, and 3 of unknown sex; Fig. 5). Ninety-two females could be assigned as mothers, accounting for 222 of the recorded births. Eighty-one out of the 92 mothers reached sexual maturity at Affenberg (*N* = 73 females born at Affenberg, *N* = 8 founder females) and gave birth for the first time there. On average, females’ age at first birth was 4.92 years (SD 0.87 years). The youngest first-time mother was 3.03 years old, and the oldest first-time mother was 7.85 years. Most females at Affenberg gave birth to their first offspring between 4 and 5 years of age. The distribution between male and female firstborn is equal (Table 1). The oldest female that gave birth was 19.3 years.

**Table 1:** Mother’s age at first birth and offspring sex

Mother’s age at first birth (years, values rounded)	No. of male offspring	No. of female offspring	No. of offspring of unknown sex	Total no. of offspring
3	0	1	0	1
4	13	14	0	27
5	17	19	2	38
6	7	5	0	12
7	0	2	0	2
8	0	1	0	1
Total	37	42	2	81

Births of females who had already reached sexual maturity (≥ 3.5 years) in 1996 were excluded (*N* = 11) due to unknown parity status before their arrival

Except for two outlier births in two different years—one born exceptionally early (February 2000) and one exceptionally late (August 2004)—the timing of the onset of the birth season ranged from mid-March to late July, with two peaks in late April and late May (Fig. 5; Table 2). The timing of birth was not influenced by the offspring’s sex (Fig. 5).

**Table 2:** Mother’s age, parity, and timing of birth

Mother's parity and age	February			March			April			May			June			July			August		
	E	M	L	E	M	L	E	M	L	E	M	L	E	M	L	E	M	L	E	M	L
Primiparous																					
3.03–4.16 years	0	0	0	0	0	0	0	1	0	2	6	11	4	4	0	0	0	0	0	0	0
4.65–5.45 years	0	0	0	0	0	0	1	4	14	7	7	3	0	1	1	0	0	0	0	0	0
5.90–7.85 years	0	0	0	0	0	1	4	0	1	1	3	2	1	2	0	0	0	0	0	0	0
Total	0	0	0	0	0	1	5	5	15	10	16	16	5	7	1	0	0	0	0	0	0
Multiparous	1	0	0	0	4	10	13	25	24	19	10	13	10	2	2	2	1	1	0	0	1
Total all births	1	0	0	0	4	11	18	30	39	29	26	29	15	9	3	2	1	1	0	0	1

In total, 81 primiparous and 138 multiparous births were recorded over 23 years. Births of unknown mothers and of females who had already reached sexual maturity at arrival in 1996 (≥ 3.5 years) were excluded due to unknown parity status. Birth records are divided into three periods per month: E: early; M: middle; L: late

**Fig. 6:**
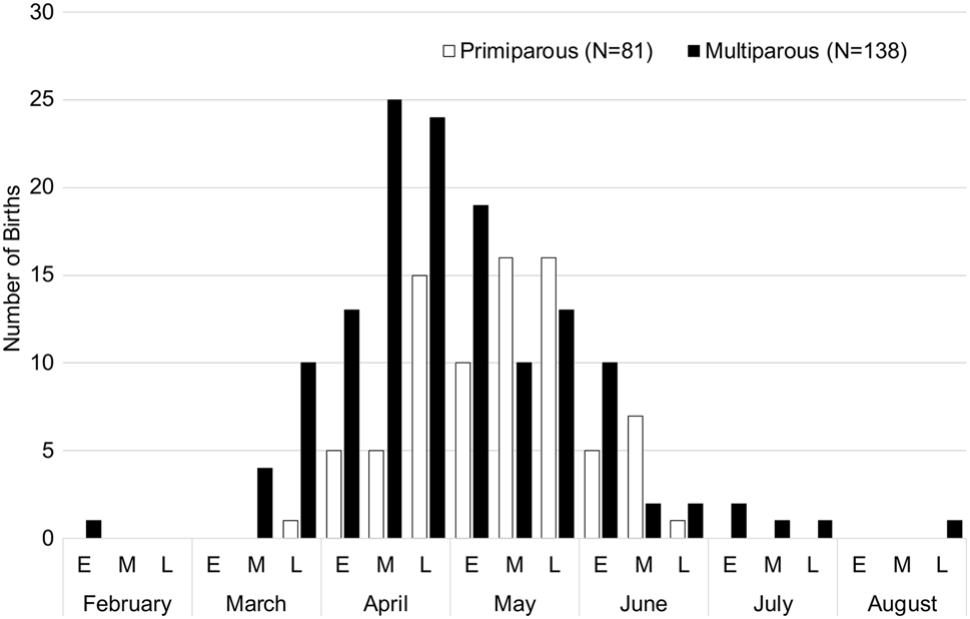
Timing of birth according to parity (*N* = 219). Each month is divided into trisections: E: early; M: middle; L: late.

Figure 6 shows the monthly distribution of 219 births according to female parity status (Table 2). Figure 6 shows no difference in timing of births between primiparous and multiparous females. Two unrelated females gave birth to their third-born exceptionally early (9 February 2000) and exceptionally late (27 August 2004) (Table 2).

#### Interbirth interval (IBI)

Between 1997 and 2019, 81 females reached sexual maturity at Affenberg and gave birth for the first time there. Of those, 50 gave birth to at least a second offspring, and 33 individuals gave birth to at least a third. The average IBI between the first and the second birth was 1.83 years (SD 0.53 years). The average IBI between second and third birth was 1.56 years (SD 0.46 years).

The overall IBI ranged from 0.88 to 4.31 years, averaging 1.68 years (*N* = 135; SD 0.62 years). The average IBI after a live-born male was 1.63 years (*N* = 71; SD 0.53 years) and after a live-born female 1.76 years (*N* = 60; SD 0.70 years). The average IBI following infant death was 1.10 years (*N* = 18; SD 0.32 years) versus 1.77 years (*N* = 117; SD 0.60 years) following a surviving infant.

#### Birth sex ratio

**Fig. 7:**
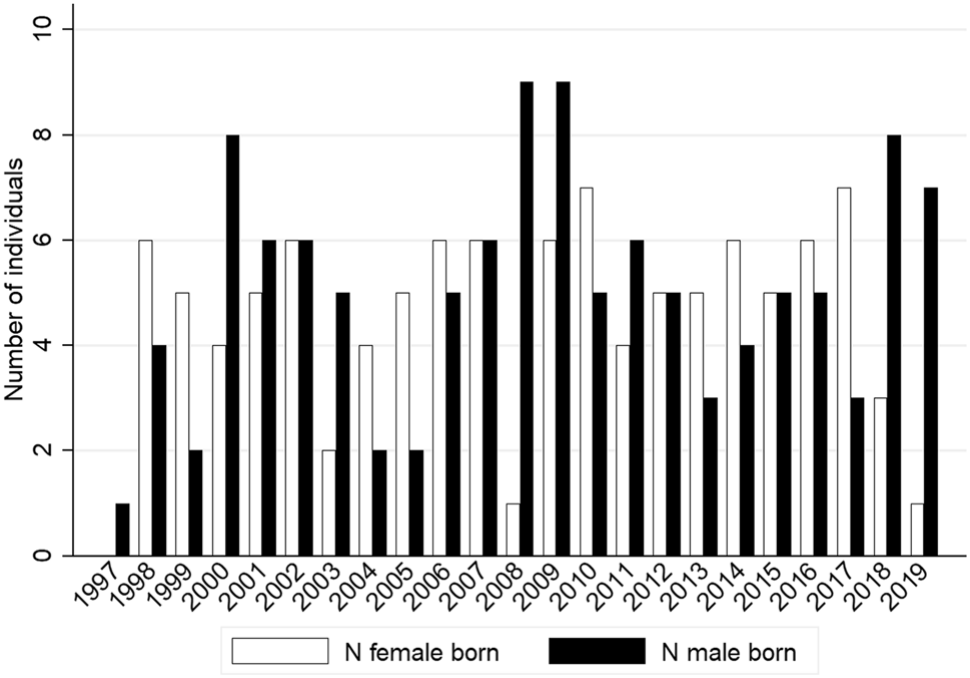
Annual variation in the number of males (*N* = 116) and females (*N* = 104) born between 1997 and 2019

In general, more females than males were born per year (Fig. 7). The sex ratio at birth (SRB) ranged from 40 to 900 (mean: 170.04; SD 215.57). The low SRB value of 40 refers to the birth seasons of 1999 and 2005, when only two males and five females were born. In 2008, nine males and only one female were born, leading to a strikingly high SRB value of 900. In only eight birth seasons were more males than females born. In 2002, 2007, 2012, and 2015, the values were equal.

#### Paternity

Using hair samples as the DNA source, paternity could be established for 43 out of 85 offspring sampled (2009–2012). The 43 offspring could be assigned to 19 fathers (out of 34 potential fathers sampled), who sired between one and five offspring each. The father’s age at infant birth ranged from 3.92 to 12.92 years (mean: 7.22 years; SD 1.78 years). Three full-blood sibling pairs were detected; three males reproduced twice with the same female.

Rank data were not available for all males who entered Radler’s (2014) paternity study. Three identified fathers holding long-tenure alpha (“Max”), beta (“Ralph”), and gamma (“Oskar”) positions, however, were identified (see “Social status” and “Stability of alpha status” sections)*.* Those males were classified as top-ranking males, the others entered the study as subordinates (*N* = 16). Due to the low number of males per rank class, only descriptive data are given here.

Ten offspring could be assigned to the three top-ranking males (alpha: *N* = 4; beta: *N* = 2; gamma: *N* = 4). The remaining 33 offspring were assigned to subordinate males. Mean age at offspring birth did not differ between top ranks (mean: 7.37 years; SD 1.48 years) and subordinates (mean: 7.22 years; SD 1.78 years), although the youngest (3.92 years) and oldest (12.92 years) detected father was a subordinate male. On average, the three top-ranking males sired slightly more offspring than subordinates (high-ranked: 3.33 offspring; SD 1.15 offspring; subordinates: 2.26 offspring; SD 1.56 offspring).

In general, males mainly reproduced during their early reproductive years (mean years between fathers’ sexual maturation and offspring births: 3.26 years; SD 1.77 years; *N* = 43). This was also the case for the three top-ranking males, who sired their offspring within their first to fifth reproductive years. For more details see electronic supplementary material Table S3.

In autumn 2003, a 2.5-year-old male, “Junior,” was transferred from an Austrian rescue center for animals to Affenberg and integrated into the group. Given the available parentage records, he fathered at least one offspring. Junior was the only individual to enter the group (other than by birth) during the 23-year period of this study. Most of the observed mating pairs were not genetically related (31 out of 34 pairs). Two pairs, however, were related, being uncle–niece and aunt–nephew, respectively. One additional pair remained unresolved because they could have been either father and daughter or half-brother and sister.

### Mortality and age range

In total, 102 deaths were recorded over the 23-year study (female = 43; male = 56; sex unknown = 3). The first death was recorded in 1997, the last in 2019. Thirty-one deaths were observed; in 58 cases a body was found shortly after death and could be identified. Only 13 individuals were declared missing. Causes of death were not always known, for example because the macaques could not be caught for medical treatment and/or their bodies were found too late to determine cause of death. Confirmed causes of death included heavy wounds and paralysis of main body parts due to accidents (in which case the animals had to be euthanized), wound infections/sepsis, blunt trauma, and cancer. According to the Affenberg staff, most of the sudden and unexpected deaths of sexually mature males (≥ 4.5 years) were, although not confirmed, the result of fatal accidents such as neck fractures after falling from a tree.

The mean age of death for females was 10.49 years (SD 8.09 years), and that for males was 9.64 years (SD 6.98 years). The oldest male and female still alive as of 1 January 2020 were 23 and 32 years, respectively.

**Fig. 8:**
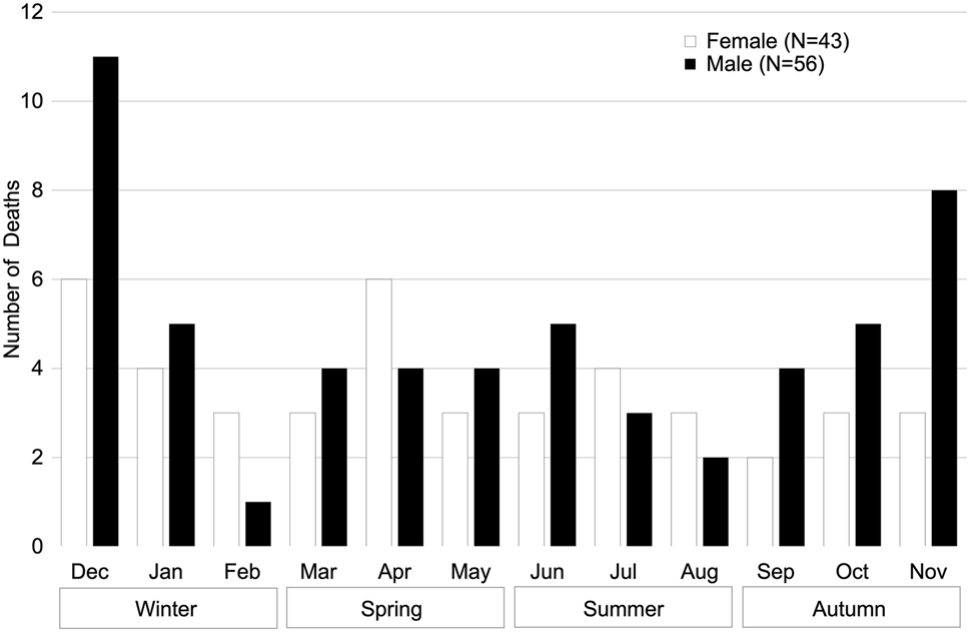
Monthly and seasonal distribution of male and female mortality

Mortality fluctuated seasonally. In total, 42.86% of male deaths occurred between October and December, peaking in December, followed by November. Among females, four months (December, January, April, July) accounted for 46.51% of the deaths, with peaks in December and April (Fig. 8).

**Fig. 9:**
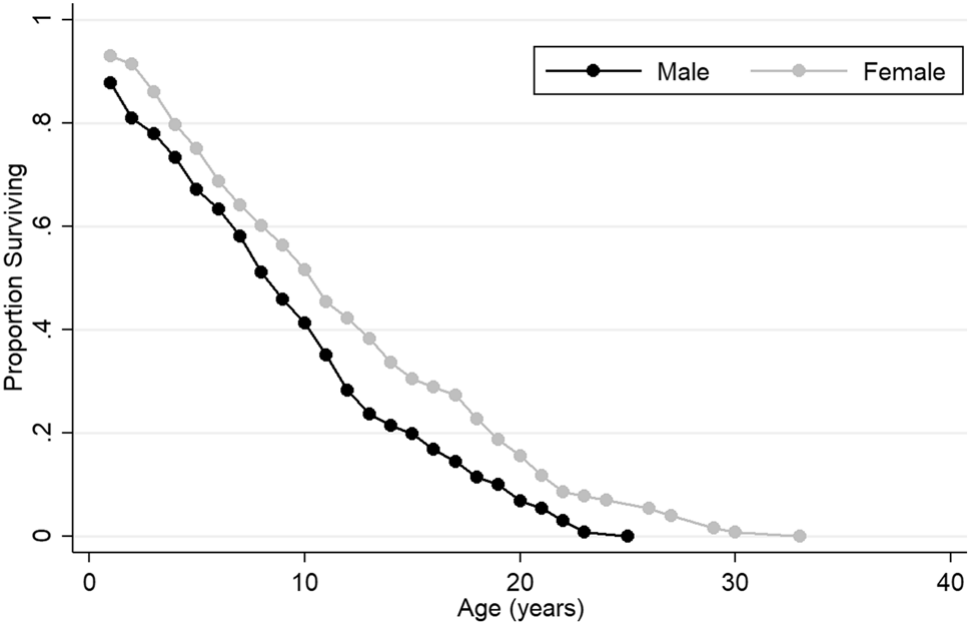
Survival curves of males and females (1996–2019)

Life tables are well established among various primate species and indicate age-specific survival rates (see electronic supplementary material Table S4). In total, 128 females and 131 males were recorded between August 1996 (arrival date) and 1 January 2020 (the cutoff point for the current study) at Affenberg. In general, the group exhibited slightly concave survival curves. The survival rates steadily decreased with age (Fig. 9).

The survival rate differed by sex. In the age group zero, the male survival rate was 87.79%, in contrast to 92.97% for females. Furthermore, the chances of a male reaching the age of 20 were 5.34%, versus 11.72% for females (see electronic supplementary material Table S4).

#### Infant mortality

Out of the 223 live-born infants, 20 died by the age of one (female = 8; male = 9; sex unknown = 3), yielding an infant mortality rate of 8.97%. Three out of these 20 infant deaths were known to be the result of kidnapping (*N* = 1 female, *N* = 1 male, *N* = 1 unknown sex) by one female, who took the newborns away from her granddaughter but was not able to lactate. The causes of 17 infant deaths remain unknown. Out of the 20 infant deaths, 13 (65%) were offspring of primiparous females and 7 (35%) were offspring of multiparous females. Out of the 20 infant deaths, 16 occurred within the first month of life. Accordingly, neonatal mortality accounted for 80% of all infant deaths. Males accounted for 50% and females for 32% of neonatal deaths.

## Discussion

Twenty-three years ago, 38 Japanese macaques were translocated from Minoo, Japan, to Carinthia, Austria. The population steadily grew, and a total of 262 individuals have lived in the group since that time. Over the past two decades, data on their reproduction, mortality, and social dynamics were continuously collected, enabling us to create a demographic profile of this Austrian population. Since their relocation from Japan, the individuals of the Affenberg population have lived semi-free in a forest area. They were not restricted in any social interactions or mating activities, nor was any individual ever removed from the group. Note, however, that captive conditions as such required provisioning, birth control, and treatment of severe injuries, and prevented males from migrating out of their natal group. We paid special attention to how these conditions affected our analyses on annual birth records, female lifetime reproductive success, and the stability of male social rank.

### Group size and composition

The study group received food on a daily basis throughout the year, and the amount given was adjusted according to the population size as it grew. In Japan, provisioning of free-ranging groups started in the 1950s (Yamagiwa 2010). Since then, the positive influence of food provisioning on survival and birth rates of Japanese macaque populations has been well documented (Koyama et al. 1992; Kurita et al. 2008). As a consequence of prolonged provisioning, the group size of free-ranging Japanese macaques has increased rapidly. Irrespective of their habitat or location, group size tends to surpass the 100-individual mark after 10–20 years of constant provisioning (Yamagiwa 2010). Despite birth control, the Austrian group reached 100 individuals a mere 12 years after their arrival on site.

According to Fedigan et al. (1983), the mean annual growth rate is about 12–14% in many provisioned groups of monkeys. In their study on the population dynamics of the Arashiyama-West group after translocation to Texas, they noted fluctuations in population growth over time. In the first 2 years following translocation, group size declined sharply due to both deaths and a poor birth rate, but quickly began to increase again, returning to a demographic pattern consistent with that of their sister group remaining in Japan. The same pattern was recognized in the Austrian group. The poor birth rate in the first year after arrival can be explained in part by the stress associated with translocation, as has been reported for other translocated groups (Fedigan et al. 1983). Environmental conditions, such as climatic parameters, could have been responsible for fluctuations in the reproductive outcome of females in the subsequent years (Pflüger et al. 2019).

The time-varying composition of the Affenberg population is comparable to that of other Japanese macaque populations such as the Arashiyama-West group both before and after their translocation to Texas (Fedigan et al. 1983). The increased number of old individuals found under semi-free conditions can be explained by reduced nutritional stress due to provisioning (Takahata et al. 1995). The use of birth control further suppressed the numbers of infants in the present study group. Since no sexually mature male was present in the translocated group, the sex ratio was low after arrival. In 1998 the SSR started to increase sharply and has never fallen below 50 since; i.e., the number of mature males has never been less than half the number of mature females. Before provisioning started in free-ranging groups of Japanese macaques in Japan, an average SSR of 65 was assessed in wild populations (Fooden & Aimi 2005; Takasaki and Masui 1984). The values, however, apparently varied from group to group; in some the number of males even exceeded that of adult females (Shodoshima-I group) or was rather low (SSR = 27.3), as was reported for the Minoo-A group back in 1984. Note here that blocked male dispersal in the present study group biased the sex ratio towards males. Although adolescent males were regularly observed to keep their distance from the main part of the group (also see “Stability of social rank” section below), they eventually became core members as they socially matured.

### Stability of social rank and paternity

Japanese macaques are categorized as one of the most despotic-nepotistic species among the genus *Macaca* (Thierry 2000). While an individual’s maternal lineage defines the hierarchical position of most females, different social circumstances can affect how a male achieves an alpha position. Such circumstances rather include the loss of the former alpha male due to death, departure, physical weakness, or group fission, than aggressive turnovers (Hayakawa and Soltis 2011; Suzuki et al. 1998; Yamagiwa 1985). Immigration and emigration influences group dominance relationships in many Old World monkey species (Sprague et al. 1998). Adult males usually join a new group at the bottom rank position of the male hierarchy because they tend to be relatively young and are not yet fully grown and/or socially matured when they immigrate. Accordingly, a male’s social rank often correlates with age, but it may decline again with advanced age or when a competitive male enters the group at a top-ranking position (Sprague 1992). In Affenberg, no migration or immigration of males is possible. Until the end of 2019, the population showed no signs of group fission, potentially supporting the stability of male alpha status. During the 23 years of our investigation, only four alpha males kept their alpha status until death or separation from the group for veterinary care. Stable dominance hierarchies, in terms of male social rank, are not uncommon in groups of Japanese macaques (Perloe 1992; Huffman 1991a) and are well described in other hierarchically organized species such as free-ranging rhesus macaques (*Macaca mulatta*; Manson 1992; Milich et al. 2020) and baboons (*Papio hamadryas ursinus*; Beehner et al. 2005).

After the loss of an alpha male in the present study group, the restructuring of the hierarchy favored the beta male, the next male in line, who had supported the former alpha for several years. Males generally do engage in male–male affiliative relationships, showing close proximity, traveling together, grooming, and agonistic support. Such male–male social bonds occur in both high-ranking and adolescent males. Detailed focal observations, however, are needed to assess the stability and strengths of these bonds and to differentiate those from taking advantage of a high-ranking supporter by forming coalitions (Kawazoe 2020). As reported in other provisioned groups of Japanese macaques (Yamagiwa 2010), high-ranking members of the Affenberg group tend to concentrate on feeding spots in the center of the group, where high-quality food such as wheat is provided. Even though adolescent males are nowadays all natal to the group, most of them make use of the peripheral area of the enclosure to keep a distance from the main group (Sprague et al. 1998). During the mating season, however, these young, low-ranking males become more present in central areas accompanied by female consort partners. As no exchange of non-troop members is possible under this closed captive condition, these adolescent males might appear attractive as “novel” mating partners, which gives them the chance to integrate into the group and eventually gain a higher status (Fujita 2010). In many primate species, females favor novel males for mating (Small 1993) and they tend to avoid breeding repeatedly with the same males (*Macaca fuscata*—Huffman 1991a,b; Inoue et al. 1990; Takahata et al. 1999; *Macaca mulatta—*Manson 1995; Nurnberg et al. 1994). This is in line with the paternity records of Affenberg, where full-blood siblings were rare, and most of the males sired offspring in their early reproductive years. This was also the case for the alpha, beta, and gamma male. We are aware that more data are needed to examine a possible relationship between male social rank and reproductive success in our study group. We assume, however, that the novelty-seeking nature of females and the stability of male rank hindered top-ranking males from maintaining high reproductive success after a certain period of being present in the group (Huffman 1991a, b, Huffman, 1992; Inoue and Takenaka 2008; Perloe 1992, also see Milich et al. 2020 for data on *Macaca mulatta*).

In free-ranging Japanese macaques, the chances of mating with close relatives are minimized because males usually leave their natal group before entering sociosexual maturity (Fooden and Aimi 2005). Furthermore, a long-term study in the Arashiyama B group revealed that close matrilineal kin dyads strongly avoided inbreeding, and in those rare cases in which it did occur, it was unlikely to result in pregnancy (Takahata et al. 2002). Even under semi-free or captive conditions, macaques tend to avoid breeding among close kin (Itoigawa, et al. 1981; Soltis et al. 1999; Widdig et al. 2017). At Affenberg, most mating partners were not kin-related, but inbreeding was not fully avoided either. The few cases of related pairs (aunt–nephew, father–daughter, or paternal half-siblings) have been, although rarely, observed mating in semi-natural groups (Inoue et al. 1990; also see Fooden and Aimi 2005 for data on natural groups). Once, a female rejection behavior was reported in the Affenberg group between a father and daughter (Radler 2014). Female macaques appear to be able to distinguish between the degrees of kinship relatedness with others due to the nepotistic matrilineal social structure, intensive early maternal investment and their strong tendency to select novel males over established ones in the group as mating partners (Takahata et al. 2002). In the abovementioned case, it remains uncertain whether the female was aware of her relationship to the male. Previous studies revealed that offspring in captivity avoided mating with individuals in close relationships with their mothers (Itoigawa et al. 1981). Kuester et al. (1994) found strong mating avoidance among co-residing maternal relatives in a Barbary macaque (*Macaca sylvanus*) population at Affenberg Salem, Germany. Free-ranging rhesus macaques also seem to discriminate paternal kin via facial cues under natural conditions (Pfefferle et al. 2014). More data on paternal kinship are needed to draw a more comprehensive picture about mate choice strategies in terms of inbreeding avoidance in the Affenberg group, and to investigate whether the rate of kin inbreeding might have changed over time in this closed group (Widdig et al. 2017).

### Birth records

A birth period ranging from spring to summer corresponds to birth records of free-ranging groups in Japan (Fooden and Aimi 2005; Itoigawa et al. 1992; Koyama et al. 1975). Those authors describe the average birth peak as being in late March, which is slightly earlier than the birth peaks in the Katsuyama and Arashiyama groups in mid/late May (Itoigawa et al. 1992). The 18 years of birth data (*N* = 430) in the Arashiyama group furthermore revealed that the birth season can continue up to mid-August. In the Affenberg group, only one exceptional birth was reported this late. Within the typical seasonal time frame of the birth season (spring–summer) in Japan, birth peaks were found to vary geographically in free-ranging Japanese macaques (Fooden and Aimi 2005). Documentation of the Arashiyama (latitude 35° 0′ 33.8112″ N; longitude 135° 40′ 1.2252″ E) monkeys translocated to Texas (latitude 28°40′2.93″ N; longitude 99°10′14.12″ W) in 1972 revealed no shift of birth season despite striking climatological and environmental differences (Gouzoules et al. 1981). However, a considerable shift occurred in a group translocated to Tasmania, in the Southern Hemisphere (Fooden and Aimi 2005). There, birth timing shifted by about 6 months. Fooden and Aimi (2005) concluded in their review that the translocation to new latitudes within the Northern Hemisphere has only minimal effects on birth timing. Accordingly, no great change after the translocation of the Minoo group to Carinthia, Austria, was expected, and none was detected. Because active birth control was performed on the Affenberg group, no female realized her lifetime natural reproductive effort. The reproductive window for females spanned on average 5 years. Accordingly, no conclusions could be drawn about whether the timing of birth varies across a female’s age and reproductive history. The oldest female to give birth was one of the original founder females born in Japan. She was 19 years old when she last gave birth, after which she was sterilized in 2000. In free-ranging provisioned groups, the oldest females reported to give birth were between 25 and 26 years old (Chalmers et al. 2012; Itoigawa et al. 1992; Koyama et al. 1992). The average age at first birth in the Affenberg group (4.9 years) was comparable to reports on provisioned groups in the wild, such as the Arashiyama (Koyama et al. 1992), Arashiyama-West (Fedigan et al. 1986), and the Katsuyama group (Itoigawa et al. 1992). In free-ranging non-provisioned Japanese macaques, the average value was reported to be higher (6.1 years in Yakushima, 7.1 years in Kinkazan; Takahata 1980). The Affenberg group was provisioned throughout the year, with the amount of food based on the nutritional need of Japanese macaques dealing with harsh winter conditions; the vegetation in the enclosure offered additional foraging opportunities. Faster maturation and hence an earlier onset of reproduction can be explained by the availability of highly nutritious foods year-round in provisioned groups. Comparable findings were reported by Mori et al. (1997) and Sugiyama and Ohsawa (1982). A similar trend can be observed in other primate species, including humans, in which girls who receive nutritional supplementation in prenatal life and/or early childhood display accelerated maturation (age at menarche) and have a faster transition to first birth (Ramakrishnan et al. 1999).

### Interbirth interval (IBI)

The literature on both provisioned and non-provisioned groups gives IBI lengths of up to 4 years (Fedigan et al. 1986; Itoigawa et al. 1992; Koyama et al. 1992). The Affenberg group conforms to this result (overall IBI ranged from 0.88 to 4.31 years). Although Itoigawa et al. (1992) found no significant differences in the average IBI between age classes of the mothers, the maximum of 4 years was reached only in females older than 15. In the Affenberg group, due to birth control, most females did not reach a high reproductive age. Here, high IBI scores of 4 years were found only in two females after their translocation from Japan to Austria. The stress of translocation and the subsequent adaptation period might explain those longer IBIs. The mean values of the present study group fit data gathered from provisioned groups such as the Arashiyama (Chalmers et al. 2012; Koyama et al. 1992) and Katsuyama groups (Itoigawa et al. 1992), but were slightly shorter than in non-provisioned free-ranging troops of Yakushima (Takahata et al. 1998) and the provisioned Arashiyama-West (Texas) group in their first 20 years after translocation (McDonald Pavelka and Fedigan 1999). The shortened birth interval in provisioned Japanese macaques is associated with the availability of highly nutritious food such as wheat (Koyama et al. 1992; Sugiyama and Ohsawa 1982). The Affenberg group contained a large number of adult females, including many older females. The presence of post-reproductive grandmothers might also lead to shorter IBI, as proposed by Pavelka et al. (2002).

Studies on free-ranging Japanese macaque (Itoigawa et al. 1992) and semi-free-ranging Barbary macaque populations (Paul and Thommen 1984) showed significantly longer IBIs between the first and second birth compared to IBIs following subsequent births. In the Affenberg group, informative data on IBIs in relation to female reproductive history were not available because of the use of birth control, sometimes applied already after a female gave birth to one offspring. The observed shorter IBI following an infant death, however, is in line with previous studies on different macaque species, such as *Macaca maurus* (Okamoto et al. 2000), *Macaca thibetana* (Zhao and Deng 1988), *Macaca fuscata* (Koyama et al. 1992), *Macaca assamensis* (Fürtbauer et al. 2010), and *Macaca cyclopis* (Hsu et al. 2001).

### Mortality

The survival curves of the Affenberg population indicate that neither males nor females are at a greater risk of dying at any time during their lives. The survival curves and the overall survivorship findings clearly contrast with the findings of Fedigan and Zohar (1997). They found that males have a significantly lower survivorship than females, and that males become more susceptible to mortality once they reach sexual maturity. They argue that the higher male mortality is due to the “high-risk, high-gain strategy.” One possible explanation why our results differ from those of Fedigan and Zohar (1997) is that young males in Affenberg cannot migrate and are therefore not exposed to the risks associated with emigration. Our findings, however, show that males are at greater risk of dying during the months of October–December. Increased aggression and involvement in conflicts among reproductively mature males might be responsible for fatal accidents during the mating season, as shown in closely related rhesus macaques (*Macaca mulatta:* Wilson and Boelkins 1970; Hoffman et al. 2008). This may also explain the rather sharp decrease in male deaths in January and February, the final 2 months of the mating season, when the secretion of male sex steroids starts to decline again (Barrett et al. 2002). In general, as food scarcity is not an issue in provisioned groups, harsh environmental conditions during winter months, social stress, and increased occurrence of injuries during the mating period potentially influence death rates in both sexes. While two months of the mating season were responsible for most female deaths, another peak occurred at the beginning of the birth season. Fedigan and Zohar (1997) also reported a higher disappearance rate for females during the birth season. They linked this to the fact that females move away from the group to give birth in a more secluded area, and if they die during parturition their bodies are not easily found. Hoffman et al. (2008) also reported higher female mortality in the birth season in a free-ranging population of rhesus macaques (*Macaca mulatta*) on the island of Cayo Santiago, Puerto Rico. These results further support the argument that reproduction comes at the expense of survival probability even in provisioned populations without predators.

The infant mortality rate in the present study group is comparable to that in other provisioned macaque species, including Japanese macaques *Macaca fuscata* (Itoigawa et al. 1992; Kurita et al. 2008), *Macaca sylvanus* (Paul and Thommen 1984), *Macaca assamensis* (Fürtbauer et al. 2010), and *Macaca thibetana* (Berman et al. 2007), but is lower in comparison with wild Japanese macaques, where infant mortality was almost three times higher (Takahata et al. 1998).

At Affenberg, most infant deaths occurred in the first month of life. This finding agrees with previous studies, in which 60–70% of the infant deaths occurred in the first 3 months (Itoigawa et al. 1992; Koyama et al. 1992; Watanabe et al. 1992). Furthermore, our data indicate that infant mortality is higher among primiparous than multiparous females (*Macaca fuscata:* Itoigawa et al. 1992; *Macaca fascicularis:* Naiken et al. 2015; *Macaca mulatta:* Bercovitch et al. 1998). This might reflect the fact that first time mothers are less experienced in rearing offspring (Sugiyama and Ohsawa 1982). Although our data point towards a tendency for males to be at a higher risk of dying within the first month of age, this sex difference could be a by-product of stochastic processes producing variations in small samples (Kurita 2010). Moreover, it is likely that not all miscarriages were recognized, and some infants might have never been found. Previous studies found no differences between mortality rates in male and female infants in provisioned macaque groups when food was abundant (Fedigan and Zohar 1997; Koyama et al. 1992; Kurita 2010).

Overall, the findings from the Affenberg Landskron group are in agreement with those of provisioned, free-ranging, semi-free-ranging, and translocated semi-free-ranging Japanese macaque populations living in the Northern Hemisphere. This attests to both the adaptability of the species and the effects of provisioning. Birth control measures obscured certain demographic parameters, and the inability of adolescent males to migrate from their natal group might be reflected in alternative social coping strategies. The formation of male coalitions and social bonds, and how they affect male social rank dynamics, clearly merits greater attention in future studies. In conclusion, the present study represents another valuable record of the situation and management success post-translocation of a primate group and provides important baseline information for future research on the Affenberg population.

## Supplementary Information

Below is the link to the electronic supplementary material.

Supplementary file1 (PDF 10414 kb)

Supplementary file2 (XLSX 24 kb)

Supplementary file3 (DOCX 18 kb)

Supplementary file4 (DOCX 18 kb)

Supplementary file5 (DOCX 20 kb)

Supplementary file6 (DOCX 18 kb)
