# Supplementary material for: Twenty-three-year demographic history of the Affenberg Japanese macaques (Macaca fuscata), a translocated semi-free-ranging group in southern Austria
Source: Primates. 2021 Jul 10;62(5):761–76. doi: 10.1007/s10329-021-00928-4 (PMC8410734; doi:10.1007/s10329-021-00928-4)
Supplement: Supplementary file 1 — Supplementary file1 (PDF 10414 kb) [file 10329_2021_928_MOESM1_ESM.pdf]

Monkey Mountain

Enclosure Orthophoto

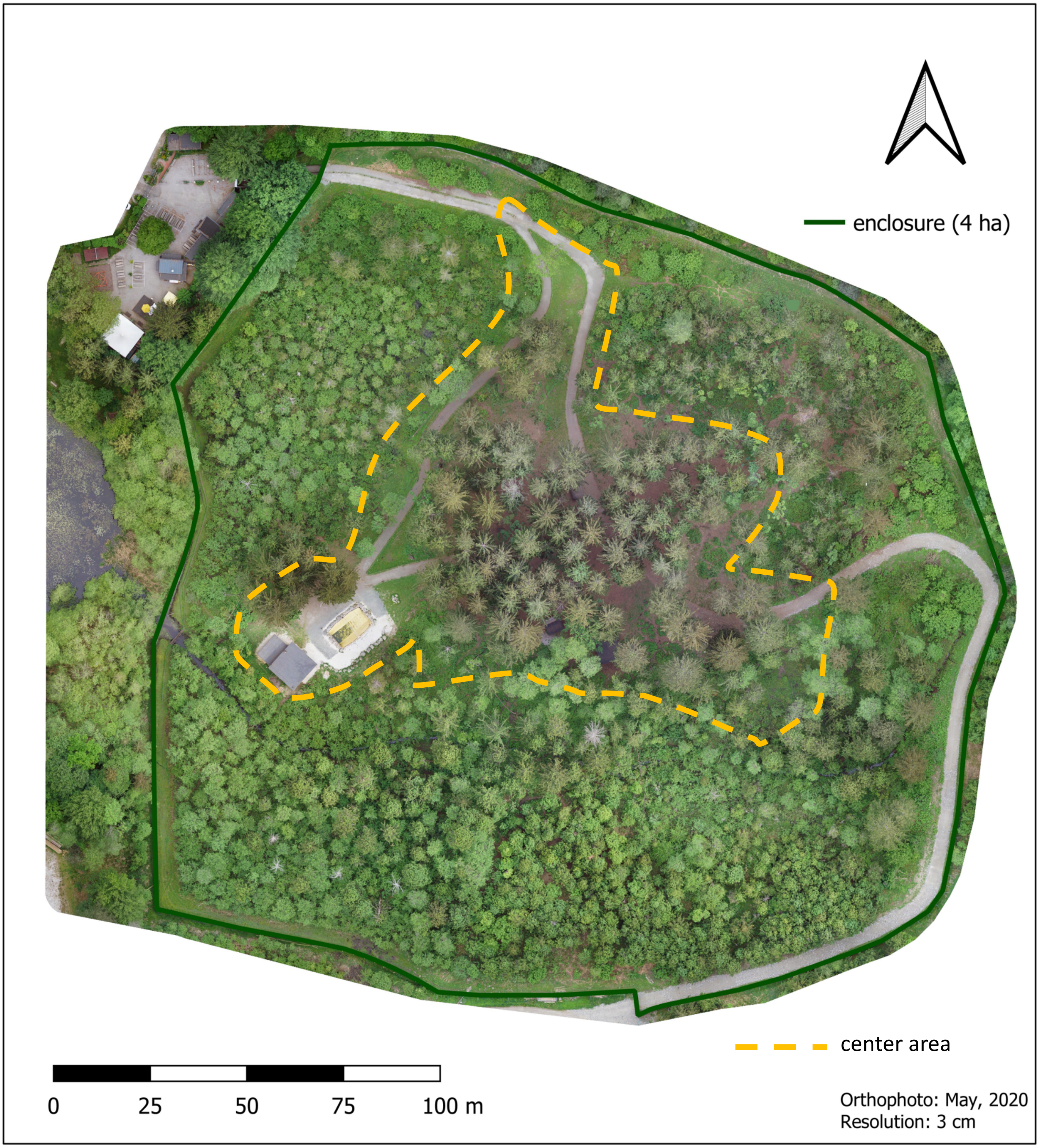

© Carinthia University of Applied Sciences – Geoinformation and Environment

Author: Alexander Lahofer, 2020
