# Supplementary material for: Twenty-three-year demographic history of the Affenberg Japanese macaques (Macaca fuscata), a translocated semi-free-ranging group in southern Austria
Source: Primates. 2021 Jul 10;62(5):761–76. doi: 10.1007/s10329-021-00928-4 (PMC8410734; doi:10.1007/s10329-021-00928-4)
Supplement: Supplementary file 3 — Supplementary file3 (DOCX 18 kb) [file 10329_2021_928_MOESM3_ESM.docx]

| **Table S1.** Annual growth rate (in %) dependent on the number of births and deaths within the foregoing annual period. | | | | |
| --- | --- | --- | --- | --- |
| **Year** | **Group size** | **Births**  **(within foregoing annual period)** | **Deaths**  **(within foregoing annual period)** | **Growth rate** |
| 16.08.96 | 38 | N/A | N/A | N/A |
| 01.09.97 | 36 | 1 | 3 | -5.26% |
| 01.09.98 | 45 | 10 | 1 | 25.00% |
| 01.09.99 | 51 | 7 | 1 | 13.33% |
| 01.09.00 | 63 | 12 | 0 | 23.53% |
| 01.09.01 | 72 | 10 | 1 | 14.29% |
| 01.09.02 | 80 | 12 | 4 | 11.11% |
| 01.09.03 | 83 | 7 | 4 | 3.75% |
| 01.09.04* | 84 | 6 + Junior | 6 | 1.20% |
| 01.09.05 | 89 | 7 | 2 | 5.95% |
| 01.09.06 | 94 | 11 | 6 | 5.62% |
| 01.09.07 | 102 | 12 | 4 | 8.51% |
| 01.09.08 | 109 | 10 | 3 | 6.86% |
| 01.09.09 | 123 | 15 | 1 | 12.84% |
| 01.09.10 | 130 | 12 | 5 | 5.69% |
| 01.09.11 | 136 | 10 | 4 | 4.62% |
| 01.09.12 | 141 | 10 | 5 | 3.68% |
| 01.09.13 | 144 | 8 | 5 | 2.13% |
| 01.09.14 | 150 | 10 | 4 | 4.17% |
| 01.09.15 | 154 | 13 | 9 | 2.67% |
| 01.09.16 | 156 | 11 | 9 | 1.30% |
| 01.09.17 | 159 | 10 | 7 | 1.92% |
| 01.09.18 | 166 | 11 | 4 | 4.40% |
| 01.09.19 | 164 | 8 | 10 | -1.20% |
| 01.01.20 | 160 | 0 | 4 | -2.44% |
| * in the year 2004, six offspring were born, and one external young male (Junior) adds to the group composition as he entered the group in the end of 2003. | | | | |
