# Supplementary material for: Twenty-three-year demographic history of the Affenberg Japanese macaques (Macaca fuscata), a translocated semi-free-ranging group in southern Austria
Source: Primates. 2021 Jul 10;62(5):761–76. doi: 10.1007/s10329-021-00928-4 (PMC8410734; doi:10.1007/s10329-021-00928-4)
Supplement: Supplementary file 4 — Supplementary file4 (DOCX 18 kb) [file 10329_2021_928_MOESM4_ESM.docx]

Table S2

| **Table S2.** Annual reproductive status of females and reproductive outcome in the subsequent birth season. Given are the number of mature females, how many of those were sterilized and resulting number and mean age of potentially fertile females measured on 1^st^ of September of each year. | | | | | | |
| --- | --- | --- | --- | --- | --- | --- |
| **Year** | **No. of mature females**  **(> 3.5 years)** | **No. of sterilized females** | **No. of potentially fertile (PF) females** | **Mean age (± SD) of PF females** | **Births in the next birth season** | **Percentage of PF females who**  **gave birth** |
| 1996 | 16 | 0 | 16 | 6.7 ± 3.4 | 1 | 6.25 |
| 1997 | 18 | 0 | 18 | 7.1 ± 3.6 | 10 | 55.56 |
| 1998 | 18 | 0 | 18 | 7.9 ± 3.7 | 7 | 38.89 |
| 1999 | 19 | 0 | 19 | 8.6 ± 3.8 | 12 | 63.16 |
| 2000 | 19 | 2 | 17 | 9.1 ± 2.9 | 10 | 58.82 |
| 2001 | 25 | 6 | 19 | 7.7 ± 3.8 | 12 | 63.16 |
| 2002 | 29 | 18 | 11 | 5.6 ± 3.8 | 7 | 63.64 |
| 2003 | 32 | 22 | 10 | 4.6 ± 1.6 | 6 | 60.00 |
| 2004 | 35 | 21 | 14 | 4.8 ± 1.8 | 7 | 50.00 |
| 2005 | 40 | 20 | 20 | 5.0 ± 1.9 | 11 | 55.00 |
| 2006 | 40 | 21 | 19 | 5.5 ± 1.3 | 12 | 63.16 |
| 2007 | 41 | 21 | 20 | 6.2 ± 1.6 | 10 | 50.00 |
| 2008 | 46 | 21 | 25 | 6.4 ± 2.1 | 15 | 60.00 |
| 2009 | 52 | 30 | 22 | 6.0 ± 2.5 | 12 | 54.55 |
| 2010 | 55 | 33 | 22 | 5.7 ± 2.5 | 10 | 45.45 |
| 2011 | 54 | 35 | 19 | 6.0 ± 2.0 | 10 | 52.63 |
| 2012 | 58 | 38 | 20 | 5.2 ± 1.5 | 8 | 40.00 |
| 2013 | 62 | 41 | 21 | 5.2 ± 1.8 | 10 | 47.62 |
| 2014 | 65 | 40 | 25 | 5.7 ± 1.9 | 13 | 52.00 |
| 2015 | 67 | 46 | 21 | 5.7 ± 2.0 | 11 | 52.38 |
| 2016 | 69 | 50 | 19 | 6.2 ± 2.5 | 10 | 52.63 |
| 2017 | 74 | 53 | 21 | 6.4 ± 2.9 | 11 | 52.38 |
| 2018 | 79 | 57 | 22 | 5.8 ± 2.5 | 8 | 36.36 |
| 2019 | 80 | 56 | 24 | 5.5 ± 2.1 | N/A | N/A |
