# Supplementary material for: Twenty-three-year demographic history of the Affenberg Japanese macaques (Macaca fuscata), a translocated semi-free-ranging group in southern Austria
Source: Primates. 2021 Jul 10;62(5):761–76. doi: 10.1007/s10329-021-00928-4 (PMC8410734; doi:10.1007/s10329-021-00928-4)
Supplement: Supplementary file 5 — Supplementary file5 (DOCX 20 kb) [file 10329_2021_928_MOESM5_ESM.docx]

| **Table S3.** Identified fathers, their year of maturation, data on their offspring, and rank classification. | | | | | | |
| --- | --- | --- | --- | --- | --- | --- |
| Father | Year of sexual maturity | Offspring | Birth year offspring | Age at offspring birth | Classfication rank | Years between sexual maturation and birth of offspring |
| Adrian | 2003 | offspring1 | 2005 | 5.87 | subordinate | 2 |
| Adrian | 2003 | offspring2 | 2007 | 7.86 | subordinate | 4 |
| Adrian | 2003 | offspring3 | 2008 | 8.94 | subordinate | 5 |
| Alois | 2006 | offspring1 | 2009 | 7.12 | subordinate | 3 |
| Alois | 2006 | offspring2 | 2009 | 7.06 | subordinate | 3 |
| Carlos | 2006 | offspring1 | 2010 | 7.92 | subordinate | 4 |
| Double-U | 2002 | offspring4 | 2002 | 3.94 | subordinate | 0 |
| Double-U | 2002 | offspring5 | 2002 | 3.92 | subordinate | 0 |
| Double-U | 2002 | offspring3 | 2005 | 6.89 | subordinate | 3 |
| Double-U | 2002 | offspring1 | 2007 | 9.07 | subordinate | 5 |
| Double-U | 2002 | offspring2 | 2011 | 12.92 | subordinate | 9 |
| Felix | 2004 | offspring1 | 2006 | 6.07 | subordinate | 2 |
| Gustav | 2006 | offspring1 | 2007 | 4.98 | subordinate | 1 |
| Herkules | 2002 | offspring2 | 2005 | 6.92 | subordinate | 3 |
| Herkules | 2002 | offspring5 | 2005 | 6.80 | subordinate | 3 |
| Herkules | 2002 | offspring1 | 2006 | 7.86 | subordinate | 4 |
| Herkules | 2002 | offspring3 | 2006 | 7.91 | subordinate | 4 |
| Herkules | 2002 | offspring4 | 2006 | 7.78 | subordinate | 4 |
| Julius | 2006 | offspring1 | 2011 | 9.07 | subordinate | 5 |
| Junior | 2005 | offspring1 | 2008 | 7.03 | subordinate | 3 |
| Ludwig | 2007 | offspring1 | 2010 | 6.96 | subordinate | 3 |
| Max | 1996 | offspring1 | 1998 | 5.90 | alpha | 2 |
| Max | 1996 | offspring4 | 1998 | 6.02 | alpha | 2 |
| Max | 1996 | offspring3 | 1999 | 6.97 | alpha | 3 |
| Max | 1996 | offspring2 | 2000 | 7.92 | alpha | 4 |
| Mickey | 2000 | offspring1 | 2001 | 4.95 | subordinate | 1 |
| Nikolaus | 2005 | offspring4 | 2008 | 7.02 | subordinate | 3 |
| Nikolaus | 2005 | offspring1 | 2009 | 7.96 | subordinate | 4 |
| Nikolaus | 2005 | offspring2 | 2009 | 8.00 | subordinate | 4 |
| Nikolaus | 2005 | offspring3 | 2010 | 9.16 | subordinate | 5 |
| Oskar | 1997 | offspring4 | 1998 | 4.98 | gamma | 1 |
| Oskar | 1997 | offspring2 | 2000 | 6.77 | gamma | 3 |
| Oskar | 1997 | offspring3 | 2001 | 7.98 | gamma | 4 |
| Oskar | 1997 | offspring1 | 2002 | 9.10 | gamma | 5 |
| Ralph | 1996 | offspring2 | 2000 | 7.95 | beta | 4 |
| Ralph | 1996 | offspring1 | 2002 | 9.90 | beta | 6 |
| Schlumpfi | 1999 | offspring1 | 2000 | 5.05 | subordinate | 1 |
| Schüchi | 1999 | offspring3 | 2001 | 6.03 | subordinate | 2 |
| Schüchi | 1999 | offspring1 | 2002 | 6.92 | subordinate | 3 |
| Schüchi | 1999 | offspring2 | 2002 | 7.07 | subordinate | 3 |
| Schüchi | 1999 | offspring4 | 2006 | 11.08 | subordinate | 7 |
| Stefan | 2005 | offspring1 | 2007 | 6.12 | subordinate | 2 |
| Willi | 1999 | offspring1 | 2000 | 4.92 | subordinate | 1 |
| Paternity analyses were conducted by Radler, 2014 between April 2009 and February 2012 using hair samples as DNA source. | | | | | | |
