# Supplementary material for: Twenty-three-year demographic history of the Affenberg Japanese macaques (Macaca fuscata), a translocated semi-free-ranging group in southern Austria
Source: Primates. 2021 Jul 10;62(5):761–76. doi: 10.1007/s10329-021-00928-4 (PMC8410734; doi:10.1007/s10329-021-00928-4)
Supplement: Supplementary file 6 — Supplementary file6 (DOCX 18 kb) [file 10329_2021_928_MOESM6_ESM.docx]

Table S4

| **Table S4** Lifetable for males and females covering the years 1996-2019. | | | | | | | | |
| --- | --- | --- | --- | --- | --- | --- | --- | --- |
| **Age group (yrs)** | **Total number of indviduals in age group** | | **Total number of deaths in the age group** | | **Survival chance in the age group** | | **Mortality rate in the age group** | |
|  | ♂ | ♀ | ♂ | ♀ | ♂ | ♀ | ♂ | ♀ |
| 0 | 131 | 128 | 16 | 9 | 0.878 | 0.923 | 0.122 | 0.070 |
| 1 | 115 | 119 | 9 | 2 | 0.809 | 0.914 | 0.078 | 0.017 |
| 2 | 106 | 117 | 4 | 7 | 0.779 | 0.859 | 0.038 | 0.060 |
| 3 | 102 | 110 | 6 | 8 | 0.733 | 0.797 | 0.059 | 0.073 |
| 4 | 96 | 102 | 8 | 6 | 0.672 | 0.750 | 0.083 | 0.059 |
| 5 | 88 | 96 | 5 | 8 | 0.634 | 0.688 | 0.057 | 0.083 |
| 6 | 83 | 88 | 7 | 6 | 0.580 | 0.641 | 0.084 | 0.068 |
| 7 | 76 | 82 | 9 | 5 | 0.512 | 0.602 | 0.118 | 0.061 |
| 8 | 67 | 77 | 7 | 5 | 0.459 | 0.563 | 0.104 | 0.065 |
| 9 | 60 | 72 | 6 | 6 | 0.412 | 0.516 | 0.100 | 0.083 |
| 10 | 54 | 66 | 8 | 8 | 0.351 | 0.453 | 0.148 | 0.121 |
| 11 | 46 | 58 | 9 | 4 | 0.282 | 0.422 | 0.196 | 0.069 |
| 12 | 37 | 54 | 6 | 5 | 0.237 | 0.383 | 0.162 | 0.093 |
| 13 | 31 | 49 | 3 | 6 | 0.214 | 0.336 | 0.097 | 0.122 |
| 14 | 28 | 43 | 2 | 4 | 0.199 | 0.305 | 0.071 | 0.093 |
| 15 | 26 | 39 | 4 | 2 | 0.168 | 0.289 | 0.154 | 0.051 |
| 16 | 22 | 37 | 3 | 2 | 0.145 | 0.273 | 0.136 | 0.054 |
| 17 | 19 | 35 | 4 | 6 | 0.115 | 0.227 | 0.211 | 0.171 |
| 18 | 15 | 29 | 2 | 5 | 0.099 | 0.188 | 0.133 | 0.172 |
| 19 | 13 | 24 | 4 | 4 | 0.069 | 0.156 | 0.308 | 0.167 |
| 20 | 9 | 20 | 2 | 5 | 0.053 | 0.117 | 0.222 | 0.250 |
| 21 | 7 | 15 | 3 | 4 | 0.031 | 0.086 | 0.429 | 0.267 |
| 22 | 4 | 11 | 3 | 1 | 0.008 | 0.078 | 0.750 | 0.091 |
| 23 | 1 | 10 | 0 | 1 | 0.008 | 0.070 | 0.000 | 0.100 |
| 24 | 1 | 9 | 1 | 0 | 0.000 | 0.070 | 1.00 | 0.000 |
| 25 | - | 9 | - | 2 | - | 0.055 | - | 0.222 |
| 26 | - | 7 | - | 2 | - | 0.039 | - | 0.286 |
| 27 | - | 5 | - | 0 | - | 0.039 | - | 0.000 |
| 28 | - | 5 | - | 3 | - | 0.016 | - | 0.600 |
| 29 | - | 2 | - | 1 | - | 0.008 | - | 0.500 |
| 30 | - | 1 | - | 0 | - | 0.008 | - | 0.000 |
| 31 | - | 1 | - | 0 | - | 0.008 | - | 0.000 |
| 32 | - | 1 | - | 1 | - | 0.000 | - | 1.00 |
